# Extrapyramidal syndrome in psychotic depression: a case report.

**DOI:** 10.1192/j.eurpsy.2023.2227

**Published:** 2023-07-19

**Authors:** C. Álvarez, A. M. Gómez Martín

**Affiliations:** Hospital Universitario Príncipe de Asturias, Alcalá de Henares, Spain

## Abstract

**Introduction:**

Psychotic depression is a subtype of major depression, with worst prognosis but underdiagnosed and undertreated. We introduce the case of a 75-year-old patient who is attended in the hospital presenting sorrow and behavioral disturbances. He also had delusions of ruin and surveillance through his phone, adding amnesia, dizziness, constipation, tremor and bradykinesia. He had suffered a limited depressive episode regarding his wife’s death.

**Objectives:**

To highlight the importance of a correct differential diagnosis in psychotic depression to prescribe an adequate treatment that provides a better outcome for the patient.

**Methods:**

A narrative search of the available literature on the subject through the presentation of a case.

**Results:**

The presumptive diagnosis is Parkinson vs psychotic depression. After some weeks of treatment with venlafaxine and olanzapine, the absence of improvement and fluctuating symptoms orientates towards Parkinson. This is later excluded due to a normal DATSCAN. Therefore, the diagnosis of psychotic depression is made, explaining parkinsonism as secondary to psychotropics. Olanzapine and venlafaxine are retired, introducing clozapine because of its lower incidence of extrapyramidal symptoms. After two weeks, the symptoms disappear, recovering the patient his basal functionality.

**Conclusions:**

Depression with psychotic symptoms can take several weeks to respond to treatment, requiring a proper organic screening. In our case, the slow response to treatment made the organic etiology as one of the main differential diagnoses, specifically Parkinson disease. It ruled out because of the absence of findings in the DATSCAN and the resolution of the extrapyramidal symptoms with the change of treatment.

**Disclosure of Interest:**

None Declared

